# Concept for Embedded Primary Care Pharmacist Practitioners (PCPPs): A Disruptive Value-Proposition

**DOI:** 10.3390/pharmacy8040195

**Published:** 2020-10-23

**Authors:** George E. MacKinnon

**Affiliations:** School of Pharmacy, Department of Family & Community Medicine, Institute for Health Equity, Genomic Sciences and Precision Medicine Center, Medical College of Wisconsin, Milwaukee, WI 53226, USA; gmackinnon@mcw.edu

The work environments and expectations for the daily activities of primary care physicians are daunting and often include spending a significant amount of time related to chronic care management with complex medication regimens, medication reconciliation, and the documentation within the electronic medical record (EMR) of these medication related issues. For a variety of reasons, there has been a reduction in physicians that are pursuing primary care roles in the United States. The Association of American Medical Colleges (AAMC) 2020 Report projected shortfalls in primary care ranging between 21,400 and 55,200 physicians by 2033 [1]. Supplanting of physicians by advance practice providers (i.e., nurse practitioners and physician assistants) will not meet the growing healthcare needs and required expertise to best serve the growing United States population. The time for the addition of an experienced healthcare provider to the primary healthcare team has come, and that provider is the pharmacist.

Given this projected shortage of physicians and the explosion of high cost specialty pharmaceuticals and growing use of biomarkers and the future of pharmacogenomics in precision medicine, the addition of a pharmacist to most physician practices will be clinically and financially prudent, if not essential in time. A future should be envisioned where pharmacists are embedded in primary care settings as primary care pharmacist practitioners (PCPPs).

As pointed out in the 2014 publication [2], physician burnout is associated with lower patient satisfaction, reduced health outcomes, and it may increase total healthcare costs. As such, dissatisfied physicians are more likely to prescribe inappropriate medications that can result in inexpensive complications [2], thus an opportunity for pharmacists to engage at the point of prescribing (e.g., in the primary care clinic setting) could provide a welcomed new member to the primary healthcare team.

Clearly, embedded pharmacists in primary and specialty care could assume other roles as called for in the 2011 U.S. Surgeon Report [3]. This call, which is now almost ten years since, has also been in part supported by the American Medical Association (AMA) as exemplified in that it has produced a tool to help physicians improve patient care called “STEPS”, which includes a relevant module (*Embedding Pharmacists Into the Practice: Collaborate with Pharmacists to Improve Patient Outcomes)* [4].

Having pharmacists embedded in primary care is not revolutionary, rather, it is evolutionary. In the United States, there are multiple models of where pharmacists are practicing within the full scope of their licensure and are included as credentialed members of the primary healthcare team. The Veterans Administration (VA), Indian Health Service (IHS), and Department of Defense (DoD) have recognized the unique and valuable contributions that pharmacists can provide to beneficiaries for the past 40 years. The Middleton VA in Madison, Wisconsin was highlighted in *USA Today* where there is one clinical pharmacist per six physicians, accounting for over 25% of patient encounters with chronic medical conditions seen by an embedded pharmacist in primary care clinics [5]. As reported, there is a desire to increase the ratio to one pharmacist to three physicians in primary care at the Middleton VA. On a commercial level, Kaiser Permanente has led this effort in the Western states for the past twenty years, and this has been occurring in other states within larger academic medical centers. Why this trend? Simply stated, chronic care management utilizes medications to treat patients, and who better to add to this team than the healthcare provider who has more education and knowledge about medications, the pharmacist?

To address these forthcoming challenges, our health profession academies, in particular medicine and pharmacy, must collectively think differently (or disruptively), but collaboratively. The described intentions are to create, in conjunction with our medical colleagues and other healthcare providers, disruptive practice models and teams of the future that leverage appropriately, so the role of the pharmacist is to achieve better health outcomes in all patients. The pharmacy profession in conjunction with medicine, needs to step forward to accept this challenge and make changes for the benefit of patients, society, and providers.

While the pharmacist workforce is well educated (at the doctoral level for the past 40 years) and highly accessible, this widely distributed group of healthcare professionals is vastly underutilized. Recent uptake and desire by patients to have immunizations provided at pharmacies by pharmacists, is a prime example of care that is convenient and cost-effective, yet delivered by another healthcare provider. As pointed out in a 2012 *New York Times* article [6], pharmacists are capable of adjusting medications, ordering and interpreting laboratory tests, and coordinating follow-up care, but state and federal laws complicate this, even though patients prefer the convenience of dealing with pharmacists.

Physicians are also recognizing the underutilized role of the pharmacist. As authored by a physician in the January 28, 2019 *New York Times* article, *The Unsung Role of the Pharmacist in Patient Health* [7] the author contends that in medicine, the focus is far too often solely on the traditional doctor/patient interaction, ignoring other practitioners (such as pharmacists) who come into contact with patients more than physicians, who can help make health care better for all.

Likewise, another physician opined in the 10 October 2018 *Forbes* article, *Can Pharmacists Help Reinvent Primary Care in the United States?* [8] that we need to bring more bright minds into medicine, but we should “not ignore the secret weapon that we already have: pharmacists.” He further asserts that in many cases, time-intensive chronic disease management, which currently lies in the hands of doctors and nurse practitioners, can be handed off “to the capable hands of pharmacists-who have a mastery of medication management, as well, as behavior change.” In summary, he postulates that by relying on pharmacists and “integrating them in our healthcare delivery models, we can provide better, more affordable, and more effective care to everyone-and potentially alleviate the looming crisis in access to primary care.”

A soon to be realized future should be envisioned where pharmacists are embedded in primary care settings as PCPPs. Having a pharmacist involved at the point-of-prescribing (i.e., in clinics) provides tremendous benefits to providers and patients alike including appropriate medication selection, adherence to therapeutic guidelines, conformance with prescription formularies, and soon, precision medicine realized through pharmacogenomics. Benefits include enhanced medication adherence, fewer adverse drug-related events, reduced inappropriate healthcare utilization (e.g., emergency room visits, hospitalizations, office visits), improved clinical outcomes, higher CMS Star Ratings, greater physician and patient satisfaction, and potentially total reduced cost of care.

Additionally, these PCPPs could extend such services in underserved rural and urban areas connected through one or two integrated EHRs, thus allowing for synchronous and asynchronous communications with multiple providers, across multiple health systems as well as with community pharmacist colleagues.

As more and more payment for health services moves from fee-for-service to “value-based”, “at-risk”, or “pay for performance (P4P)” contracting, it is incumbent that health systems, physicians, and payers need to look at the full complement and shear number of healthcare providers available to meet the needs of society. Certainly, the COVID-19 pandemic of 2020 has brought forward the challenges of an overwhelmed healthcare system and need to embrace all essential healthcare providers, and that embrace needs to include pharmacists. Thought of in another way (from the patient perspective), we need to consider seeing the right patient, by the right provider, in the right place, for the right price, and where appropriate, the use of the right pharmaceutical: “the 5Ps”. I subscribe that in fact, pharmacists with physicians can help address these 5Ps in primacy care.

These articles in PHARMACY’s Special Issue “Embedded Pharmacists in Primary Care” highlight such advancements of models that have included pharmacists. These contributions span academic medical centers to family medicine training programs in both urban and rural settings as well as performing roles in chronic disease management, comprehensive medication management, and the use of collaborative practice agreements. As Guest Editor to this Special Issue, I hope you enjoy these contributions and that they inspire you to replicate these works by contacting these authors or making your own contribution in the near future!

Be well in this unprecedented times.
